# Molecular Advances in Microbial Metabolism

**DOI:** 10.3390/ijms24098015

**Published:** 2023-04-28

**Authors:** Rosa María Martínez-Espinosa, Carmen Pire

**Affiliations:** 1 Department of Biochemistry, Molecular Biology, Edaphology and Agricultural Chemistry, Faculty of Science, University of Alicante, Carretera San Vicente del Raspeig s/n, 03690 San Vicente del Raspeig, Alicante, Spain; carmen.pire@ua.es; 2 Multidisciplinary Institute for Environmental Studies “Ramón Margalef”, University of Alicante, 03690 San Vicente del Raspeig, Alicante, Spain

Climate change, global pollution due to plastics, greenhouse gasses, or heavy metals among other pollutants, as well as limited natural sources due to unsustainable lifestyles and consumption patterns, are revealing the need for more research to understand ecosystems, biodiversity, and global concerns from the microscale to the macroscale. Biochemical cycles (the cycling of atoms and molecules between living and non-living things) have been significantly affected during recent decades due to anthropogenic activities closely connected to pollution, climate change, and global warming thus affecting the quality of the air, soil, and water worldwide.

Within this context, biogeochemical cycles important to living organisms include the water, nitrogen, sulfur, carbon, and phosphorus cycles. Microbial metabolism is one of the main driving forces behind the development and maintenance of these cycles and consequently of the biosphere. Most of the main metabolic pathways of the biogeochemical cycles directly depend on microorganisms. The idea of this Special Issue arises due to the relevance of these metabolic pathways and the lack of knowledge on the molecular types of machinery making them possible. Thus, this Special Issue focuses on molecular mechanisms underlying microbial metabolism, not only to improve the knowledge of the processes carried out by microbes to obtain energy and nutrients to live and reproduce but also to shed light on microbial evolution, changes in microbial communities due to modified environmental parameters, and potential applications of metabolic pathways carried out by microbes in biotechnology and industrial processes.

Considering all these topics, the Special Issue was conceived as a multidisciplinary topic comprising several disciplines, such as microbiology, molecular biology, genetics, chemistry, microbial ecology, biochemistry, biophysics, and all omics-based sciences which offer insights into the impact that modern technologies have on microbiological research today. The Special Issue was open for submission for less than one year, and eight of the manuscripts received were finally accepted for publication, all of them original research works related to microorganisms (belonging to the bacteria domain) of relevance for biotechnological processes or human/animal diseases.

Related to the global concern about pollution by plastics and microplastics, several research groups worldwide are looking for biopolymers showing similar physicochemical characteristics to those characterizing the most marketed plastics produced from petroleum. The advantage of these biopolymers is that they are 100% recycled and even biodegradable. As a consequence of this new and innovative approach, microorganisms (including extremophiles) able to produce polyhydroxyalkanoates (PHAs) are being used as model organisms to produce bioplastics on a large scale. The work here presented by Lascu and co-workers focused on the characterization of PHA production by the bacterium *Photobacterium ganghwense* C2.2. thanks to a wide range of approaches and methods (from the genome to the bioreactor). The most significant results displayed in this work demonstrate that this bacterium can produce PHA using urea and crude glycerol as N and C sources, respectively, without pH adjustment during the fermentation which contributes to the upscaling of the process [1].

In connection with relevant industrial processes related to food processing, probiotics foods, or the use of *L. lactis* as a delivery vehicle for vaccines or to overproduced marketed compounds (i.e., antibiotics), two of the published works in this Special Issue are focused on *Lactococcus lactis* and *Bifidobacterium bifidum*, respectively [2,3]. The work addressed by Kosiorek and co-workers studied the potential influence of plasmidic genes on overall gene expression in industrially important *L. lactis* strains [2]. The main findings from this work revealed, in concordance with other previous studies in other bacteria, that the presence of plasmids in L. lactis determines relevant changes in the host phenotype and the expression of chromosomal genes. The authors suggest that the maintenance of the plasmids leads to significant perturbation in global gene regulation that affects central metabolic pathways and adaptive responses of *L. lactis* cells. This evidence should be considered in the future to improve the efficiency of all the industrial processes in which *L. lactis* strains are used in food processing, fermentations, or probiotics. On the other hand, the work carried out by Liu and co-workers performed an integration of transcriptome and metabolome to identify the genes and metabolites involved in *Bifidobacterium bifidum* biofilm formation. *B. bifidum* is a probiotic that has been used as a major ingredient to produce nutraceuticals [3]. Most of the strains can form biofilms on abiotic surfaces, thus increasing self-resistance. Despite being one of the most used bacteria for industrial purposes, little is known about the molecular mechanism of *B. bifidum* biofilm formation. Liu and coworkers devised an experimental approach by combining transcriptome sequencing and untargeted metabolomics analysis of both *B. bifidum* biofilm and planktonic cells. The aim was to identify key genes and metabolites involved in biofilm formation. The results indicate that quorum sensing, a two-component system, and amino acid metabolism are essential during *B. bifidum* biofilm formation which contributes to the management of the cells during their use in food and probiotic-based industries.

*Prevotella* has been the model genus in two of the works published in this Special Issue. Schleicher and coworkers observed that the vaginal microbiome dominated by lactobacillus is replaced by a mixed bacterial population including *Prevotella bivia* [4]. *P. bivia* is associated with bacterial vaginosis, and consequently connected with a variety of health abnormalities, such as preterm births, increased susceptibility to sexually transmitted pathogens, or a higher risk of pelvic inflammatory disease. The authors aimed to understand why and how this bacterium success replaced *Lactobacillus* populations. The main results obtained revealed that central carbon metabolism, sodium-motive electron transfer, and ammonium formation by *P. bivia* are essential molecular processes, explaining the efficiency of *P. bivia* within the vaginal microbiome. On the other hand, Trautmann and coworkers have explored the molecular mechanisms of the rumen bacteria *Prevotella bryantii* B14 for resistance to monensin (ionophore for monovalent cations, which is frequently used to prevent ketosis and to improve performance in dairy cows) [5]. The results confirmed that the high abundance and activity of the Na+-translocating NADH:quinone oxidoreductase counteracted the sodium influx caused by monensin. Moreover, cell membranes and extracellular polysaccharides were significantly influenced by monensin. Therefore, in the presence of monensin, *P. bryantii* cells perform a reconstruction of extracellular polysaccharides to survive, which consequently hinders the substrate binding capacities of this rumen bacterium.

Regarding agricultural practices and nitrogenous gas emissions from soils subjected to excessive use of fertilizers, two articles based on the soybean endosymbiont *Bradyrhizobium diazoefficiens* explain molecular mechanisms involving the role of FixK_2_ and NnrR proteins in denitrification in general (anaerobic respiratory pathway in which oxygen is replaced by nitrate or nitrite by final electron acceptors), and particularly in N_2_O turnover. Parejo and co-workers describe a fine-tuning modulation of oxidation-mediated posttranslational control of the FixK_2_ factor in vivo [6]. Bueno and coworkers reported that FixK_2_ and NnrR are regulatory determinants essential for denitrification in this bacterium [7]. Furthermore, the authors demonstrate that N_2_O reduction by *B. diazoefficiens* is independent of canonical inducers of denitrification, such as the nitrogen oxide NO_3_^−^, and it is negatively affected by extreme values of pH (both acidic and alkaline conditions).

Finally, Paula Soares and co-workers described the transcriptomic response of the bacterium *Gluconacetobacter diazotrophicus* to iron limitation [8]. This acid-tolerant microorganism is endophytic and colonizes internal plant tissues, establishing a symbiotic relationship with its host. Therefore, the optimal growth of *G. diazotrophicus* cells contributes to the health of the host. The results showed that genes encoding functions related to iron homeostasis were significantly upregulated under iron limitations, whilst certain genes involved in the secondary metabolism were overexpressed under iron-limited conditions. In contrast, the expression of genes involved in Fe-S cluster biosynthesis, flagellar biosynthesis, and type IV secretion systems was downregulated in an iron-depleted culture medium. All of this corroborates the essentiality of this micronutrient for both plant and *G. diazotrophicus* cells.

## References

[1] Lascu I., Tănase A.M., Jablonski P., Chiciudean J., Preda M.I., Avramescu S., Irgum K., Stoica I. (2022). Revealing the phenotypic and genomic background for PHA production from rapeseed-biodiesel crude glycerol using *Photobacterium ganghwense* C2.2. Int. J. Mol. Sci..

[2] Kosiorek K., Koryszewska-Bagińska A., Skoneczny M., Stasiak-Różańska L., Aleksandrzak-Piekarczyk T. (2023). The presence of plasmids in *Lactococcus lactis* IL594 determines changes in the host phenotype and expression of chromosomal genes. Int. J. Mol. Sci..

[3] Liu Z., Li L., Fang Z., Lee Y., Zhao J., Zhang H., Chen W., Li H., Lu W. (2021). Integration of transcriptome and metabolome reveals the genes and metabolites involved in *Bifidobacterium bifidum* biofilm formation. Int. J. Mol. Sci..

[4] Schleicher L., Herdan S., Fritz G., Trautmann A., Seifert J., Steuber J. (2021). Central carbon metabolism, sodium-motive electron transfer, and ammonium formation by the vaginal pathogen *Prevotella bivia*. Int. J. Mol. Sci..

[5] Trautmann A., Schleicher L., Pfirrmann J., Boldt C., Steuber J., Seifert J. (2021). Na+-coupled respiration and reshaping of extracellular polysaccharide layer counteract monensin-induced cation permeability in *Prevotella bryantii* B14. Int. J. Mol. Sci..

[6] Parejo S., Cabrera J.J., Jiménez-Leiva A., Tomás-Gallardo L., Bedmar E.J., Gates A.J., Mesa S. (2022). Fine-tuning modulation of oxidation-mediated posttranslational control of *Bradyrhizobium diazoefficiens* FixK2 transcription factor. Int. J. Mol. Sci..

[7] Bueno E., Mania D., Mesa S., Bedmar E.J., Frostegård A., Bakken L.R., Delgado M.J. (2022). Regulation of the emissions of the greenhouse gas nitrous oxide by the soybean endosymbiont *Bradyrhizobium diazoefficiens*. Int. J. Mol. Sci..

[8] Soares C.P., Trada-Sfeir M.Z., Terra L.A., Ferreira J.P., Dos-Santos C.M., Oliveira I.G.B., Araújo J.L.S., Meneses C.H.S.G., de Souza E.M., Baldani J.I. (2022). Transcriptomic response of the diazotrophic bacteria *Gluconacetobacter diazotrophicus* Strain PAL5 to iron limitation and characterization of the fur regulatory network. Int. J. Mol. Sci..

